# Characterization of SIRT1/DNMTs Functions and LINE-1 Methylation in Patients with Age-Related Macular Degeneration

**DOI:** 10.3390/jcm8020159

**Published:** 2019-02-01

**Authors:** Andrea Maugeri, Martina Barchitta, Matteo Fallico, Niccolò Castellino, Michele Reibaldi, Antonella Agodi

**Affiliations:** 1 Department of Medical and Surgical Sciences and Advanced Technologies “GF Ingrassia”, University of Catania, 95123 Catania, Italy; andreamaugeri88@gmail.com (A.M.); martina.barchitta@unict.it (M.B.); agodia@unict.it (A.A.); 2 Department of Ophthalmology, University of Catania, 95123 Catania, Italy; matteofallico@hotmail.com (M.F.); ncastellino7@gmail.com (N.C.)

**Keywords:** retinal degeneration, AMD, DNA methylation, DNMT, SIRT1, LINE-1

## Abstract

Previous studies proposed the application of DNA methylation signatures as clinical biomarkers of age-related macular degeneration (AMD). However, the characterization of Long Interspersed Nuclear Element-1 (LINE-1) methylation levels—a surrogate marker of global DNA methylation—in AMD patients has not been investigated so far. In the present study, we first characterized DNA methyltransferases (DNMTs) and Sirtuin 1 (SIRT1) functions in blood samples of 40 AMD patients and 10 age- and sex-matched controls. Then, we evaluated whether changes in DNMTs functions were associated with different LINE-1 methylation levels in leukocyte DNA. We demonstrated that total DNMTs activity was 48% higher in AMD patients than in controls (*p* = 0.005). AMD patients also exhibited up-regulation of DNMT1 and DNMT3B expression (FC = 2.6; *p* = 0.003 and FC = 2.4; *p* = 0.018, respectively). In line with increased DNMTs functions, the LINE-1 methylation level was higher in AMD patients than in controls (mean = 69.10%; SE = 0.68 vs. mean = 65.73%; SE = 0.59; *p* = 0.020). All *p*-values were adjusted by Bonferroni correction. In AMD patients, LINE-1 methylation level was positively associated with total DNMTs activity (*r* = 0.694; *p* < 0.001), DNMT1 (*r* = 0.579; *p* < 0.001), and DNMT3B (*r* = 0.521; *p* = 0.001) expression. Our results encourage further large-size prospective research to understand the relationship between LINE-1 methylation and AMD aetiology, and its usefulness in the clinical setting.

## 1. Introduction

Age-related macular degeneration (AMD) is a neurodegenerative disease that leads to the progressive destruction of the neurosensory macular area, involving retinal pigment epithelium (RPE), Bruch’s membrane and choroid [1]. According to the severity of symptoms, the disease is classified into early, intermediate, and advanced stages. While the early stage is characterized by the aberrant pigmentation of the RPE and the accumulation of “drusen” (i.e., yellowish lipid-rich, protein-containing extracellular deposits accumulating between RPE and Bruch’s membrane), the advanced stage may manifest as non-exudative or exudative AMD. Approximately 170 million individuals are affected by AMD worldwide, with a prevalence that ranges from 2% to 20% among elderly people. AMD is thereby the leading cause of blindness in developed countries and the third leading cause globally [2,3]. Several lines of evidence suggest a possible role for epigenetic changes—including DNA methylation, histone modification, and expression of non-coding RNA—in AMD etiology. Aberrant epigenetic patterns have also been described in several pathophysiological processes—such as aging, oxidative stress, inflammation, and angiogenesis—that are related to the pathogenesis of retinal degenerative diseases [4,5,6,7]. Previous epidemiological studies observed the dysregulation of the S-adenosylmethionine cycle in AMD patients, which in turn contributes methyl donors for DNA methylation [8]. In mammals, the methylation process almost always occurs at short DNA sequences (i.e., CpG islands), which typically contain around 5-10 CpGs per 100 bp. The methylation process is carried out by DNA methyltransferases (DNMTs), out of which only DNMT1, DNMT3A and DNMT3B are catalytically active [9]. Recently, it has been demonstrated that the activity of DNMT1—the enzyme responsible for maintenance of DNA methylation—is regulated by Sirtuin 1 (SIRT1) [10], a NAD+-dependent histone deacetylase with multiple roles in aging, apoptosis, DNA repair, inflammation, and oxidative stress [11]. Up to 80% of CpG islands is localized in non-coding regions scattered throughout the genome that mainly contribute to the global methylation status [9]. Long Interspersed Nuclear Element-1 (LINE-1) sequences, accounting for ≈18% of human genome, have been widely used as a surrogate marker of global methylation in aging and age-related disease [12,13,14,15]. In the context of AMD, previous studies have focused on DNA methylation in genes involved in the etiology of the disease [16,17,18,19]. Instead, the characterization of LINE-1 methylation levels in AMD patients has not been investigated so far. In the present study, we first characterized DNMTs and SIRT1 functions in blood samples of AMD patients and age- and sex-matched controls. Then, we evaluated whether changes in DNMTs functions were associated with different LINE-1 methylation levels in leukocyte DNA.

## 2. Materials and Methods

### 2.1. Study Design

From July 2017 to July 2018, subjects that had been referred to the Department of Ophthalmology, University of Catania (Italy), were enrolled in the present study. The study protocol was approved by the ethics committee of the involved institution and performed according to the Declaration of Helsinki. Subjects were fully informed of the purpose and procedures of the study and an informed consent was signed. Since we aimed at exploring changes in LINE-1 methylation levels between AMD patients and controls, we calculated a priori sample size for Student’s *t*-test using PS power and sample size program (version 3.0) [20]. Based on a hypothetical difference of 1.0 ± 1.0% 5-mC in LINE-1 methylation levels between cases and controls, the sample size of 50 subjects (4:1 ratio between cases and controls) was required to reach a statistical power of 80% with a significance level of 0.05.

During the routine eye exam, AMD cases were selected by dilated retinal exam, optical coherence tomography, and fluorescein angiography. The cases involved naïve patients diagnosed with wet AMD and scheduled for intravitreal anti vascular endothelial growth factor agent injection, aged over 50 years, with no history of ocular diseases of the posterior segment, except of AMD. Individuals with cardiovascular disease, diabetes mellitus, autoimmune disorders, and history of cancer were excluded. Age- and sex-matched AMD-free controls were enrolled among patients who underwent cataract surgery fulfilling the inclusion and exclusion criteria. Information on sociodemographic and lifestyle characteristics were collected by trained epidemiologists using a structured questionnaire. Educational level was classified as low (≤8 years of school) and high (>8 years of school). Subjects were also classified as employed or unemployed (including students and housewives). Their BMI was calculated as weight (kg) divided by height (m^2^), based on criteria from the World Health Organization [21]. For smoking status, the subjects were classified as either, non-smokers (including ex-smokers) and current smokers. From each participant, a peripheral blood sample was collected into Ethylenediaminetetraacetic acid (EDTA) tubes for molecular analysis.

### 2.2. DNMTs and SIRT1 Activity Quantification

Nuclear proteins were extracted from peripheral blood samples using the Nuclear Extraction Kit according to the manufacturer’s instructions (Abcam plc, Cambridge, UK). Nuclear protein quantification was performed by the Qubit fluorometer (Invitrogen, Carlsbad, CA, USA), using the Qubit Protein Assay Kit according to the manufacturer’s instructions. The total DNMTs activity was quantified using the colorimetric DNMTs Activity Quantification Kit (Abcam plc, Cambridge, UK) according to the manufacturer’s instructions. Optical density (OD) was read within 2–10 min at 450 nm, with an optional reference wavelength of 655 nm. SIRT1 activity was quantified using the fluorometric SIRT1 activity assay kit (Abcam plc, Cambridge, UK) according to the manufacturer’s instructions. Fluorescence intensity was measured at Ex/Em= 350–450 nm for 30 to 60 min at 1–2 min interval. The total DNMTs and SIRT1 activities were reported as percentage of control.

### 2.3. DNMTs and SIRT1 Expression Analysis

The total cellular RNA was extracted using Trizol® Reagent (Invitrogen, Carlsbad, CA, USA) and reverse transcribed to single-stranded cDNA using the SuperScript III Reverse Transcriptase (Applied Biosystems, Foster City, CA, USA), according to the manufacturer’s protocols. Gene expression was determined by qPCR with TaqMan Gene Expression Assays (Life Technologies, Monza, Italy) using the QuantStudio™ 7 Flex System (Applied Biosystems, Foster City, CA, USA). Specific primers were used to detect DNMT1 (assay no. Hs00945875_m1), DNMT3A (Hs01027162_m1), DNMT3B (Hs00171876_m1), and SIRT1 (Hs01009006_m1). Data were normalized to the values of GAPDH expression (Hs02758991_g1). Relative RNA quantification was performed using the 2-ΔΔCT method [22] and reported as fold change (FC) of controls.

### 2.4. DNMTs and SIRT1 Expression Analysis

The peripheral blood samples were centrifuged at 2500 rpm for 15 min. The buffy coat fraction was transferred to a cryovial and immediately frozen at −20 °C until use. Leukocyte DNA was extracted using the QIAamp DNA Mini Kit (Qiagen, Milan, Italy) according to the manufacturer’s protocol. As previously reported [23,24], LINE-1 methylation level was measured by pyrosequencing-based methylation analysis of three CpG islands (GenBank Accession No. X58075), using the PyroMark Q24 instrument (Qiagen, Italy). Briefly, bisulfite conversion and clean-up of DNA for methylation analysis of 30–40 ng of DNA were completed using the EpiTect Bisulfite Kit (Qiagen, Italy) and the converted DNA was eluted in 20 μl of Eluition Buffer. PCR was conducted in a reaction volume of 25 μL, using the PyroMark PCR Kit (Qiagen, Italy). Each reaction mixture contained 1.5 μL of bisulfite-converted DNA, 12.5 μL of PyroMark PCR Master Mix 2X, 2.5 μL of Coral Load Concentrate 10X, and 2 μL of the forward primer (5′-TTTTGAGTTAGGTGTGGGATATA-3′) and the reverse-biotinylated primer (5′-biotin-AAAATCAAAAAATTCCCTTTC-3′) (0.2 μM for each). HotStart PCR cycling conditions were 1 cycle at 95 °C for 15 min, 40 cycles at 94 °C for 30 s, 50 °C for 30 s, and 72 °C for 30s, and a final extension at 72 °C for 10 min. Then, the PCR product underwent pyro-sequencing using 0.3 mM of the sequencing primer (5′-AGTTAGGTGTGGGATATAGT-3′). All runs included 100%, and 0%, methylated human DNA as positive, and negative controls, respectively. To confirm reproducibility every sample was tested two times and failed assays were repeated. Overall, intra-observer coefficient of variability between the two replicates of LINE-1 methylation measurements was 2.2% (SD = 1.0%). For each CpG island, LNE-1 methylation levels was calculated as %5-mC over the total of cytosines. LINE-1 methylation level was reported as the mean of the three CpG sites.

### 2.5. Statistical Analysis

The data were analyzed using GraphPad Prism (version 6.0; GraphPad Software Inc., San Diego, CA, USA) and SPSS software (version 20.0; SPSS Inc., Chicago, IL, USA). The participants’ characteristics were described using frequency (%) or mean and standard error (SE). The Kolmogorov-Smirnov test was used to test the normal distribution of continuous variables. Continuous variables underlying normal distribution were compared between AMD patients and controls, using the Student’s *t*-test followed by Bonferroni correction. The adjusted *p*-values were obtained by multiplying the unadjusted *p*-value by the number of comparisons. Linear regression analysis was used to investigate the associations of LINE-1 methylation levels with DNMTs functions. All statistical tests were two-sided, and the *p*-value < 0.05 was considered statistically significant.

## 3. Results

### 3.1. Characteristics of Study Population

Overall, 40 AMD patients (mean age = 69.8 years; 50% male) and 10 age- and sex-matched controls (mean age = 68.9 years; 50% male) were enrolled in the present study. The comparison of sociodemographic characteristics (i.e., educational level and employment status), BMI, and smoking status between AMD patients and controls revealed no significant differences (*p*-values > 0.05).

### 3.2. DNMTs and SIRT1 Functions in AMD Patients 

**Figure 1:**
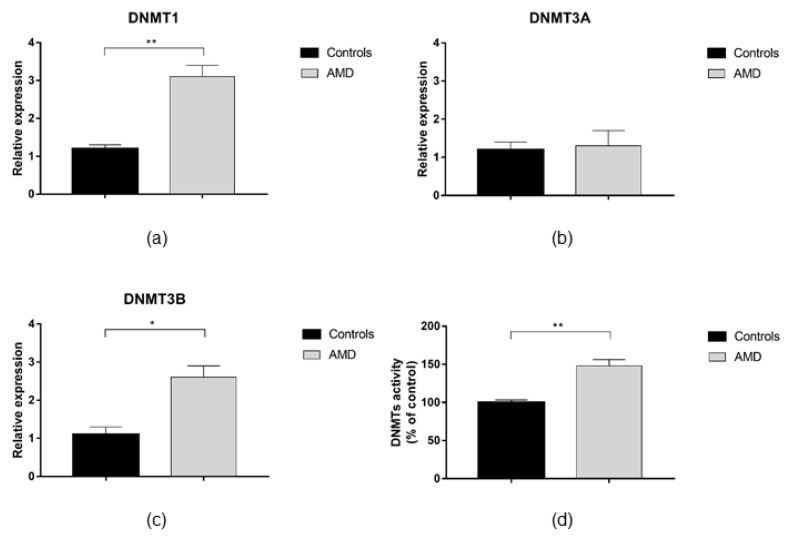
DNA methyltransferases (DNMTs) expression and activity in age-related macular degeneration (AMD) patients and age- and sex-matched controls. The analysis of gene expression showed that: (**a**) DNMT1 (*p* = 0.003) and (**c**) DNMT3b (*p* = 0.018) were up-regulated in AMD patients (*n* = 40) compared with controls (*n* = 10). (**b**) No difference was observed in DNMT3a expression. (**d**) The analysis of total DNMTs activity showed higher levels in AMD patients than in controls (48%; *p* = 0.005). Bar graphs show mean ± SE. * *p* < 0.05, ** *p* < 0.01 versus controls based on the Student’s *t*-test. DNMT, DNA methyltransferases; AMD, age-related macular degeneration; SE, standard error.

**Figure 2:**
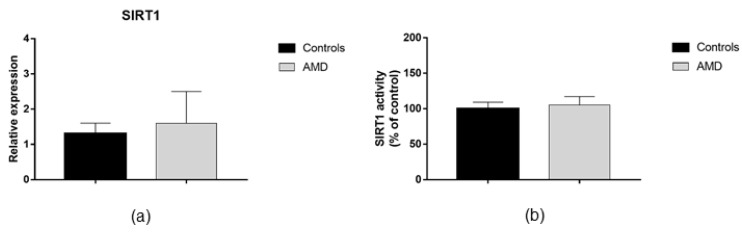
Sirtuin 1 (SIRT1) expression and activity in AMD patients and matched controls. The analysis of gene expression (**a**) and (**b**) enzymatic activity showed no differences between AMD patients (*n* = 40) and controls (*n* = 10). SIRT1, Sirtuin 1.

AMD patients exhibited up-regulation of DNMT1 and DNMT3B expression compared to healthy peers (FC = 2.6; *p* = 0.003 and FC = 2.4; *p* = 0.018, respectively; Figure 1a,c). In contrast, no difference in DNMT3A expression was evident (Figure 1b). In line with increased DNMTs expression, we demonstrated that total DNMTs activity was 48% higher in AMD patients compared with controls (*p* = 0.005; Figure 1d). Since DNMTs functions might be regulated by SIRT1 expression and activity, we also analyzed whether AMD patients exhibited altered SIRT1 functions. However, we did not find any statistically significant differences in SIRT1 expression and activity between AMD patients and controls (Figure 2).

### 3.3. LINE-1 Methylation in AMD Patients

**Figure 3:**
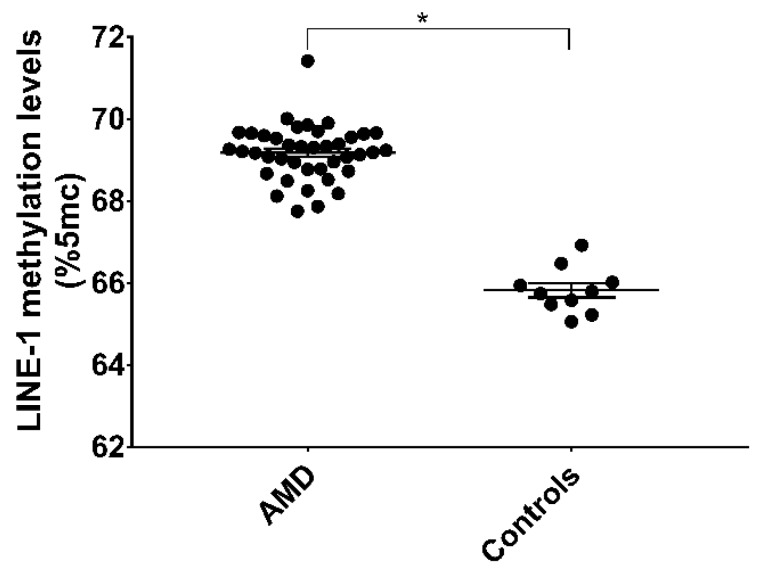
LINE-1 methylation levels in AMD patients (*n* = 40) and age- and sex-matched controls (*n* = 10). * *p* < 0.05 vs. controls based on the Student’s *t*-test. LINE-1, Long Interspersed Nuclear Element-1.

**Figure 4:**
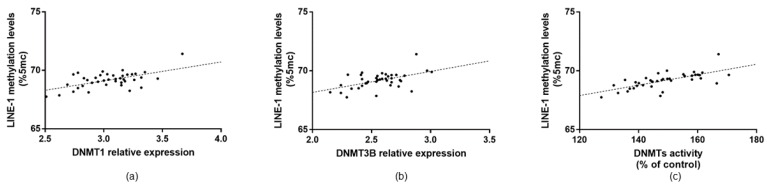
Linear regression analysis of LINE-1 methylation level with DNMTs functions. (**a**,**b**) LINE-1 methylation level was positively associated with DNMT1 (*r* = 0.579; *p* < 0.001) and DNMT3B (*r* = 0.521; *p* = 0.001) expression. (**c**) In line with increased gene expression, LINE-1 methylation level was also positively associated with total DNMTs activity (*r* = 0.694; *p* < 0.001). LINE-1, Long Interspersed Nuclear Element-1; DNMT, DNA methyltransferases.

To evaluate the effect of increased DNMTs function on global DNA methylation, we measured methylation levels of LINE-1, a widely used surrogate marker of global DNA methylation. Overall, the LINE-1 methylation level was 68.42% (SE = 0.63) and there were no differences in age, sex, educational level, employment status, BMI, and smoking status were evident (*p*-values > 0.05). In line with increased DNMTs functions, LINE-1 methylation level was higher in AMD patients compared with controls (mean = 69.10%; SE = 0.68 vs. mean = 65.73%; SE = 0.59; *p* = 0.020) (Figure 3). In AMD patients, LINE-1 methylation level was positively associated with total DNMTs activity (*r* = 0.694; *p* < 0.001). In line with the positive association with total enzymatic activity, LINE-1 methylation level was also positively associated with DNMT1 (*r* = 0.579; *p* < 0.001) and DNMT3B (*r* = 0.521; *p* = 0.001) expression (Figure 4). In contrast, no associations with DNMT3A and SIRT1 were evident.

## 4. Discussion

The present study, to our knowledge, is the first that characterises DNMTs/SIRT1 functions and LINE-1 methylation levels in peripheral blood samples from AMD patients. Previous studies have focused on DNA methylation changes in several pathways that are related to the pathogenesis of AMD. A genome-wide methylation study by Hunter and colleagues investigated DNA methylation profiles in post-mortem RPE and choroid from AMD patients. The authors found that two glutathione S transferase isoforms (i.e., GSTM1 and GSTM5) underwent epigenetic repression via hypermethylation of their promoters [16]. The epigenetic down-regulation of these detoxification enzymes in AMD patients increased their susceptibility to oxidative stress [25]. More recently, in vitro studies demonstrated that the cellular redox state of the retinal cells modulated the activation of SIRT1 [6,7], which in turn triggered the hypoxia and angiogenesis pathways via the upregulation of HIF-2α, VEGF and Erythropoietin [26]. Wei and colleagues analysed genome-wide differences in peripheral blood mononuclear cells from one pair of monozygotic twins and two pairs of dizygotic twins with discordant AMD phenotypes [17]. Notably, they reported lower methylation level within the promoter region of IL17RC in AMD patients compared with their healthy peers. The hypomethylation of its promoter enhanced the expression of IL17RC and increased the chronic inflammatory response in the macula [17]. Although these findings were further validated, either in discordant siblings for AMD, and in a traditional case-control study [17], others failed in confirming this evidence. Oliver and colleagues in fact concluded that *IL17RC* hypomethylation in peripheral blood was not a suitable clinical biomarker of AMD, highlighting the need for considerable replication of epigenetic association studies prior to clinical application [18]. Recently, it has also been proposed that hypomethylation within the promoter of the Clusterin gene—one of the major component of drusen—might be an epigenetic hallmark of AMD in the retina [19]. The hypomethylation of its promoter led to the up-regulation of Clusterin gene expression in cultured RPE cells, which were derived from AMD patients compared with age-matched healthy donors [19].

Efforts to understand the epigenetic hallmarks of AMD have led us to explore DNA methylation processes in RPE cells upon oxidative and inflammatory conditions, two of the major causes of retinal degeneration [27]. Our in vitro studies demonstrated that oxidative stress and inflammation decreased DNMTs/SIRT1 functions and LINE-1 methylation level in RPE cells [6,7]. A previous study partially confirmed this evidence, suggesting that SIRT1 expression decreased in an age-dependent manner in RPE cells and retina with AMD [28]. Next, Oliver and colleagues identified differential DNA methylation in the blood and retina of AMD patients, encouraging further studies to investigate the true concordance between blood DNA methylation and those of the RPE and retina [29]. Therefore, we first characterized DNMTs and SIRT1 functions in blood samples from AMD patients and age- and sex-matched controls. Contrary to what we observed in RPE cells, AMD patients exhibited increased total DNMTs activity, dependent of DNMT1 and DNMT3B over-expression. This controversy further supported the notion that DNA methylation profiles in leukocytes might differ from those observed in the retina. In line with the hypothesis that global DNA methylation might be modulated by a DNMTs-dependent pathway, we also demonstrated that LINE-1 methylation level was higher in blood leukocyte DNA of AMD patients than in healthy controls. Consistently, the LINE-1 methylation level was positively associated with total DNMTs activity, DNMT1 and DNMT3B expression in AMD patients. In contrast, no associations with DNMT3A and SIRT1 were evident. 

LINE-1 methylation has been widely used as a surrogate marker of global methylation in aging and age-related disease [9,10,11,12], but none of the previous studies have focused on AMD. Although there is no evidence on AMD patients, our results are in line with those reported by the research on Alzheimer disease (AD) [30,31]. For instance, Bollati and colleagues demonstrated that LINE-1 methylation level was higher in blood samples of AD patients compared with healthy volunteers [31]. Notably, AMD and AD share several environmental risk factors (e.g., smoking, systemic hypertension, and hypercholesterolemia) and histopathologic features (e.g., the deposition of amyloid-β in ocular drusen and senile plaques) [32], which might partially explain similar LINE-1 methylation changes. 

Our study has some limitations. The study design does not allow an understanding of changes in LINE-1 methylation are a cause or a consequence of AMD. Another weakness is the small sample size of our analysis, which included 40 cases and 10 age- and sex-matched controls. However, prior to analysis, we calculated a priori sample size for Student’s t-test to reach a statistical power of 80% with a significance level of 0.05. Although, control subjects were patients who underwent cataract surgery, to our knowledge, no association of DMNTs function and LINE-1 methylation with cataract has previously been reported. Previous studies showed that LINE-1 methylation levels might vary across CpG sites and different tissue [33], discouraging the comparison of our results in the context of previous researches [34]. Recently, it has also been reported that differences in blood cell composition might affect DNA methylation levels [35]. We analysed LINE-1 methylation in leukocyte DNA, but the differences in sub-type composition should be considered in future research [36]. Finally, although we used age- and sex-matched controls, we cannot rule out the influence of environmental exposure [37], lifestyles [23] and genetic variants [38,39] that might affect LINE-1 methylation and/or AMD pathogenesis. 

In conclusion, we characterized DNMTs and SIRT1 functions in blood of AMD patients, showing an increased total DNMTs activity dependent of DNMT1 and DNMT3B over-expression. In line with increased DNMTs functions, we demonstrated for the first time that LINE-1 methylation levels were higher in AMD patients compared with age- and sex-matched controls. However, further large-size prospective research is encouraged to understand the relationship between LINE-1 methylation and AMD aetiology, and its usefulness in the clinical setting.
